# MiR-29/Hsp47 in ECM network

**DOI:** 10.18632/oncoscience.227

**Published:** 2015-09-05

**Authors:** Ren Xu

**Affiliations:** Markey Cancer Center and Pharmacology and Nutritional Sciences, University of Kentucky, Lexington, KY 40536, USA

**Keywords:** extracellular matrix, microRNA-29, Hsp47

Cancer development and progression require extensive reorganization of extracellular matrix [1, 2]. ECM is a complex mixture of structural proteins, glycoproteins, and proteoglycans, which provide not only essential physical scaffolds to maintain tissues structure but also various biochemical signals to modulate cellular function. Altering the fine balance of ECM signal is sufficient in the long run to induce breast cancer development and progression. Although ECM has been identified as a determinant factor in the tumor microenvironment that controls cancer development and progression, but how ECM microenvironmental cues integrate at the genome level to promote breast cancer progression remains to be determined.

Gene co-expression network analysis is a systems biology method using correlation statistics as pairwise similarity measurements between gene expression profiles to illustrate the strong relationship which connects transcripts’ regulatory patterns to the functional organization of the cell [3]. Using gene co-expression network analysis of more than 700 human breast cancers, we have showed that ECM microenvironment is regulated at the transcription network level during breast cancer development [3]. Importantly, we have identifed Hsp47 as a hub of the ECM transcription network genes in breast cancer tissue [4]. Hsp47 is a protein chaperon and facilitates folding and secretion of collagen in the endoplasmic reticulum. Hsp47 gene locates at 11q13, a region that is often amplified in cancer tissue. Increased Hsp47 expression is associated with high cancer stages and short recurrent-free survival. We demonstrate that Hsp47 promotes tumor growth and invasion by enhancing ECM deposition in breast cancer cells [4]. These results indicate that Hsp47, possibly through its regulation of ECM network, is crucial for cancer progression and may represent a potential biomarker and therapeutic target.

Another important finding from the co-expression network analysis is the negative association between miR-29b/c levels and the ECM network gene expression in human breast cancer tissues. MiR-29 has recently been identified as a tumor suppressor by altering tumor microenvironment [5]. MiR-29 binding sites are enriched in the 3′UTR regions of the ECM network genes. Moreover, introduction of miR-29 mimics significantly reduces the expression of Hsp47 and other ECM network genes [4]. These results suggest that miR-29 is a critical negative regulator of ECM microenvironment and represses ECM protein expression at the transcription network level. Given the therapeutic potential of microRNA, identifying roles for miR-29 in regulating the ECM network may lead to discovery of a novel target for the inhibition of ECM-dependent cancer progression.

Stromal cells have been considered major source of ECM proteins. However, our and others’ studies demonstrate that cancer cells are an active and important component in ECM remodeling, and that this process is required for cancer progression and metastasis. Cancer cells are an important source of ECM in cancer tissue and deposit a significant amount collagen, fibronectin, and tenascin-C [6]. However, roles of cancer cell–produced ECM in the cancer progression have not been appreciated until recently. We show that breast cancer cells express multiple ECM transcription network genes. Introducing miR-29 or silencing Hsp47 in breast cancer cells suppresses malignant phenotypes in breast cancer cell lines by reducing collagen and fibronectin deposition. In addition, silencing Col4A1 or FN1 in breast cancer cells represses malignant phenotypes in 3D cultures [4]. These results suggest that cancer cell-deposited ECM proteins are crucial for cancer progression. Although cancer cells and stromal cells in breast cancer tissue both generate significant amount of ECM proteins, the roles of these proteins in cancer progression may be different. It has been shown that LAMA4 produced by cancer cells governs primary and metastatic tumor re-initiation [7]. Our unpublished data demonstrate cancer cell–produced ECM proteins enhance dissemination and survival of tumor cells during the early phases of metastasis. These experiments provide strong evidence that ECM molecules generated by cancer cell promote cancer progression through enhancing cell survival and proliferation as essential components of the cancer cell niche.

## References

[1] Zhu J (2014). Histology and histopathology.

[2] Bissell MJ (2011). Nature medicine.

[3] Xu R (2011). Integr Biol (Camb).

[4] Zhu J (2015). Cancer Research.

[5] Chou J (2013). Nature cell biology.

[6] Naba A (2012). Mol Cell Proteomics.

[7] Ross JB (2015). Nature cell biology.

